# Methodological challenges in a study on falls in an older population of Cape Town, South Africa

**DOI:** 10.4314/ahs.v17i3.35

**Published:** 2017-09

**Authors:** Sebastiana Z Kalula, Monica Ferreira, George H Swingler, Motasim Badri, Avan A Sayer

**Affiliations:** 1 Division of Geriatric Medicine, The Albertina and Walter Sisulu Institute of Ageing in Africa, University of Cape Town, South Africa; 2 International Longevity Centre-South Africa, Faculty of Health Sciences, University of Cape Town, South Africa; 3 Department of Paediatrics and Child Health, Faculty of Health Sciences, University of Cape Town, South Africa; 4 Department of Medicine, Faculty of Health Sciences, University of Cape Town, South Africa, and King Saud bin Abdulaziz University for Health Sciences, Riyadh, Saudi Arabia; 5 MRC Lifecourse Epidemiology Unit, Faculty of Medicine, University of Southampton, United Kingdom

**Keywords:** Falls, older people, community-based research, low and middle income countries, methodology, study design

## Abstract

**Background:**

Falls are a major cause of disability, morbidity and mortality in older persons, but have been under researched in developing countries.

**Objective:**

To describe challenges encountered in a community-based study on falls in a multi-ethnic population aged ≥65 years in a low-income setting.

**Methods:**

The study was conducted in four stages: A pilot study (n=105) to establish a sample size for the survey. An equipment validation study (n=118) to use for fall risk determination. A cross-sectional baseline (n=837) and a 12-month follow-up survey (n=632) to establish prevalence and risk factors for falls.

**Results:**

Prevalence rate of 26.4% (95% CI 23.5–29.5%) and risk factors for recurrent falls: previous falls, self-reported poor mobility and dizziness were established. Adaptations to the gold standard prospective fall research study design were employed: 1) to gain access to the study population in three selected suburbs, 2) to perform assessments in a non-standardised setting, 3) to address subjects' poverty and low literacy levels, and high attrition of subjects and field workers.

**Conclusion:**

Studies on falls in the older population of low- to middle-income countries have methodological challenges. Adaptive strategies used in the Cape Town study and the research experience reported may be instructive for investigators planning similar studies in such settings.

## Introduction

Falls are common, and a major cause of disability, morbidity and mortality in older persons^1^–^4^. A fall may affect an older individual's functioning and well-being in multiple ways, such as impaired functioning, fear of falling again^5^–^7^, and premature admission to a nursing home^8^–^9^. Even in the absence of injury, a fall may have a negative psychological impact on an older person.

Studies on falls in older persons have mainly been conducted in high-income countries^10^, and scant attention has been paid to the problem in low- and middle-income countries^11^. Studies in community settings in the former countries have reported at least one fall a year in 30 to 60% of persons aged >65 years, increasing to 50% in the age group 80 years and over^12^–^19^. In general, a gold standard prospective research design has been employed in these studies^20^, but its use may not be feasible in low- to middle-income country settings.

Despite potentially serious consequences of falls for older persons, little is known of the extent and gravity of the problem in sub-Saharan countries. The study conducted in a community based older population of Cape Town, South Africa sought to establish a prevalence rate and to identify risk factors for falls.

For reasons such as a scarcity of resources and a socio-economically diverse study population, a number of methodological challenges were anticipated and indeed encountered. The challenges and how they were overcome in the study are described in the paper.

## The South African setting

Although the United Nations lists South Africa as a middle income country^21^, the population, totalling some 52.5 million, is heterogeneous and largely poor. In 2013, the population aged >65 years numbered some 2.7 million and represented 5 per cent of the total population. By 2050, this population is projected to reach 5.7 million and to represent 10 per cent of the total population^22^.

Historically, people in the different ethnic groups resided in segregated residential areas. Although the Group Areas Act (No. 41 of 1950)^23^ was repealed in 1988, residential suburbs remain racially segregated with a socio-economic divide persisting across suburbs. Black Africans have historically been most disadvantaged on virtually all indicators, with whites most advantaged, and coloureds (people of mixed ancestry) and Indians falling in-between^24^.

## Methods

### Study design

The study design was a descriptive and analytic cross-sectional survey with a 12-month follow-up survey (2009–2010). A reassessment of subjects a year after baseline enabled additional investigation of the association between risk factors and falls.

**Figure 1:**
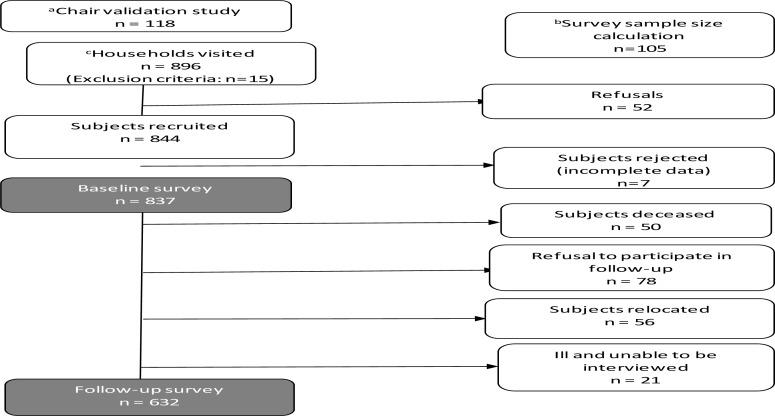
Study procedure and sample recruitment ^a^Sub-study to validate equipment (chair). Subjects were not part of the survey. ^b^Sub-study to estimate sample size, Subjects were not part of the main study. ^c^Number of households visited for the baseline survey. The required sample size was 840; 896 households with a person aged ≥ 65 years were visited and 52 refused to participate in the study. The number of subjects recruited was 844 ofwhom7were excluded due to incomplete data. The baseline survey included 837 subjects. Of these, 632 participated in the follow-up survey

The study was designed and executed in four stages, in anticipation of methodological challenges: 1) A pilot study to establish a prevalence rate for falls in order to calculate a sample size for the baseline survey. 2) A validation study of non-standardised equipment for assessment of muscle power and balance. 3) A baseline survey. 4) A 12-month follow-up survey. (See Figure1.) Ethics approval to conduct the study was obtained from the University of Cape Town.

### Sampling

Three suburbs of Cape Town were purposively selected as a sampling frame: Plumstead (with a majorly white population), Wynberg Central (with a majority coloured population) and Gugulethu (with a majorly black African population). According to the 2001 population census data, the most recent data available at the time, each of the suburbs had a high proportion of persons aged > 65 years: Plumstead: 15 per cent; Wynberg: 11 per cent: and Gugulethu: 5.6 per cent^25^. The Population Census Office does not release data on individual citizens, and a multistage sampling technique had to be used to recruit subjects based on the Office's division of suburbs into units referred to as Enumeration Areas (EAs). All households in an Enumeration Area were visited in order to increase the probability of finding a subject who met the study criteria.

Several challenges emerged in the recruitment of a sample in the three suburbs. In the case of Gugulethu, entryto the community had to be negotiated with community leaders. In the case of Plumstead, a large number of residents, mainly whites, refused to admit a field worker into the dwelling for fear of their personal safety.

The numbers and percentage distribution of study participants by suburb are shown in Table 1. The ethnic distribution was as follows: Black Africans: 283 (33.8%); coloureds: 392 (46.8%); and whites: 140 (16.7%). Twenty two (2.6%) subjects, mainly recruited in Wynberg, were Indians; these subjects were too few for independent analyses. The other sub-samples provided for comparative analysis of three ethnic data sets. In South Africa, ethnicity, or race group, remains a proxy for socio-economic status, by and large, which may impact or modify risk factors for falls and fall outcomes.

**Table 1:** Baseline survey sample, by suburb and gender: numbers and percentage distribution

	Males		Females		Total	
Suburb	n	%	n	%	n	%
Gugulethu	52	24.6	231	36.1	283	33.7
Plumstead	43	21.8	145	22.7	188	22.5
Wynberg	102	51.8	264	41.3	366	43.7
Total	197	100	640	100	837	100

Gender distribution differed by suburb, χ^2^ p=0.018

### Four stages of the study

The design and execution of the four stages of the study, and methodological challenges encountered and how they were overcome in each stage are described below.

### Pilot study to calculate a sample size

A pilot study was conducted to establish a fall prevalence rate to inform the sample size for the baseline survey. Residents aged > 65 years were randomly recruited in each of the three suburbs: seven in Gugulethu, 16 in Plumstead and 82 in Wynberg (a total of 105 subjects). Informed consent to participate in the study was obtained from each subject. The subjects were interviewed and physical measurements were performed by trained field workers in a subject's dwelling. Recruitment of this sample was not without challenges. Protracted negotiations were required with community leaders to obtain permission to work in Gugulethu which delayed the execution of the pilot study; only a small number of subjects were eventually recruited in that suburb

### A study to validate performance assessment equipment

Conventionally, lower limb strength and balance as determinants of a risk of falling are measured at a standardised central venue where a standard chair can be used to conduct physical performance tests. The resource poor environment in which the study was conducted and a limited research budget made it unfeasible to secure such a venue in each suburb as well as to transport subjects, some frail, to a central venue. The performance of physical measurements in subjects' dwellings was neither without challenges. Field workers typically lacked private transport and experienced logistical difficulties in travelling to some dwellings. They were neither able to carry heavy or bulky equipment with them to conduct tests.

A need arose therefore to source and use easily transportable measurement tools which still met specifications for various tests. A collapsible canvas chair was sourced and validated to ensure its use, as opposed to the use of a “standard” chair, would not affect the outcome of tests such as the Timed Up and Go (TUG) test. The standard chair for testing lower limb strength is an office chair with a 46 cm sitting height^26^. The collapsible chair (weight 4 kg, sitting height of 46 cm) used had an aluminium frame, a firmly fitted canvas seat and back, a straight back rest, and padded arm rests. One hundred and eighteen subjects were recruited at a retirement centre and completed the timed Up and Go test on the collapsible chair and a standard chair. A detailed description of the chair and its use has been reported elsewhere^27^.

### The baseline survey

The baseline survey was publicised in the study suburbs before hand to facilitate recruitment of a sample. Nonetheless, in Plumstead a larger than envisaged number of dwellings had to be visited to realise a sample, which ultimately, only represented 67 per cent of the required sub-sample (n=188).

The study questionnaire, to be used in a multi-ethnic sample, was translated from English into *isiXhosa*, the language spoken by the majority of black Africans in Cape Town, and back-translated. Field workers were trained in administration of the questionnaire in two languages. Several dwellings of subjects were small and cramped, and the field workers often had to re-arrange furniture to create sufficient space to perform physical assessments. They frequently had to contend with onlookers among co-inhabitants and neighbours, as well as interjections from them during interviews and physical assessments. High attrition among field workers, often due to frustration in recruiting subjects, required periodic replacement of field workers and subsequent training.

### The follow-up survey

Subjects who participated in the baseline survey were revisited approximately 12 months later, specifically to record fall occurrences and outcomes in the previous year. The low literacy level of numerous subjects and subjects' variable access to a telephone meant that neither written nor telephonic reporting of fall events could be recorded between baseline and follow-up, as prescribed in the gold standard prospective research design.

### Data management and statistical analysis

All fixed item responses were coded, and open ended responses were categorised and coded. Data codes were entered into a Microsoft Access 2003 database and data were analysed using the SPSS software (IBM SPSS Inc., Chicago Illinois). The prevalence and incidence of falls were calculated with a 95% confidence interval (CI). For continuous variables, data were tested for normality of distribution using the Shapiro-Wilks test. Data were compared using the χ 2 test or the Mann-Whitney test, as appropriate. Relationship between falls and potential predictors of recurrent falls was determined using a backward stepwise logistic regression analysis with a probability of 0.05 for a variable to enter the model and 0.10 for removal. Strength of association was expressed as an odds ratio with 95% confidence intervals (CIs). All tests were two-sided and a p-value less than 0.05 was considered significant.

### Results

The mean (SD) age of subjects in the pilot study sample was 75.7 (6.99) years. Seventy five (71%) were female. Twenty five (23.8%) reported falling at least once and eight (7.6%) reported falling more than twice in the previous 12 months. With a fall prevalence rate of 23.8%, the required sample size for the baseline survey was accordingly calculated at 280, at 80% power and p=0.0528. To allow for comparison between the three ethnic sub-samples, a sample of 840 was required (280 for each suburb) for the baseline survey.

One hundred and eighteen subjects completed the TUG test in the chair validation study. No difference was found in time taken to complete the test in the standard chair and the test chair (median (inter-quartile range) = 12.3 (9.53–15.9) and 12.6 (9.7–16.6) seconds for the standard chair and the test chair, respectively (p-value=0.87)). Use of the collapsible canvas chair was therefore deemed to offer an acceptable alternative in assessments of fall risk using the TUG test. The use of the collapsible chair was thus an improvisation that facilitated conducting such a study in a non-standardised setting.

The sample for the baseline survey comprised 837 subjects. More than three-quarters (76.5 %) were female (640 women, 197 men) (mean (SD) age 74 (6.4) years) (Table 1). Prevalence rates of 26.4% (95% CI 23.5–29.5%) for falls and 11% (95% CI 9–13%) for recurrent falls were established at baseline. Fall rates in the ethnic sub-samples differed: whites: 42.9%; coloureds: 34.4%; and black Africans: 6.4% (p=0.0005).

Only six hundred and thirty two subjects (485 women and 147 men), 76.6% of the sample of 837, could be traced and re-assessed. The mean (SD) age was 75 (6.2) years. Of the 205 subjects lost to follow up, 78 refused to participate, 56 had relocated, 21 were too ill to be interviewed and 50 had died (Figure 1). Lower prevalence rates of 21.9% (95% CI 18.9–25.3%) for falls and 6.3%up. (95% CI 4.6–8.3%) for recurrent falls were established at follow-up. Fall incidence rates of 556, 405 and 111/1000 person years were established for whites, coloureds and black Africans, respectively.

Tables 2 and 3 show univariate analyses of self-reported factors, measurements and assessment at baseline and their association as risk factors for recurrent falls at follow-up.

**Table 2:** Predictors for falls at baseline and recurrent falls status at follow-up

Characteristic	Single or no fall N=592	Recurrent falls N=40	P Value
*Residence (N (%))* Gugulethu Plumstead Wynberg	178 (98.9) 134 (89.9) 280 (92.4)	2 (1.1) 15 (10.1) 23 (7.6)	0.002*
*Previous fall (N (%))* No Yes	453 (98.1) 139 (81.8)	9 (1.9) 31 (18.2)	<0.001*
*Age Group (years) (N (%))* 65 – 69 70 – 74 75 – 79 80^+^	183 (95.3) 167 (92.8) 138 (93.9) 104 (92.0)	9 (4.7) 13 (7.2) 9 (6.1) 9 (8.0)	0.651
*Marital Status (N (%))* Married Not married	193 (96.5) 399 (92.4)	7 (3.5) 33 (7.6)	0.047*
*Ethnic group (N (%))* Black African Coloured White	179 (98.4) 315 (91.8) 98 (91.6)	3 (1.6) 28 (8.2) 9 (8.4)	0.009*
*Educational level (N (%))* No schooling Primary Secondary Post school qualification	23 (88.5) 9170 (6.6) 362 (93.3) 37 (88.1)	3 (11.5) 6 (3.4) 26 (6.7) 5 (11.9)	0.111
*Occupational category (N (%))* Unskilled Skilled Professional	204 (95.3) 321 (93.9) 67 (88.2)	10 (4.7) 21 (6.1) 9 (11.8)	0.086
*Self-rated health (N (%))* Good/very good Average/poor/very poor	316 (92.9) 276 (94.5)	24 (7.1) 16 (5.5)	0.416
*Perceived health compared to a year ago (N (%))* Better Same/worse	542 (95.1) 50 (80.6)	28 (4.9) 12 (19.4)	<0.001*
*Self-rated mobility (N (%))* Independent Impaired	522 (95.3) 70 (83.3)	26 (4.7) 14 (16.7)	<0.001*
***Self-reported medical conditions (N (%))***			
Hypertension No Yes	202 (94.0) 390 (93.5)	13 (6.0) 27 (6.5)	0.834
Stroke No Yes	550 (94.5) 42 (84.0)	32 (5.5) 8 (16.0)	0.003
Parkinson's disease No Yes	583 (93.9) 9 (81.8)	38 (6.1) 2 (18.2)	0.150
Diabetes No Yes	450 (93.4) 142 (94.7)	32 (6.6) 8 (5.3)	0.566
Memory loss No Yes	558 (93.6) 34 (94.4)	38 (6.4) 2 (5.6)	0.844
Depression No Yes	537 (94.7) 55 (84.6)	30 (5.3) 10 (15.4)	0.002
Arthritis No Yes	227 (91.9) 365 (94.8)	20 (8.1) 20 (5.2)	0.144
Chronic lung disease No Yes	535 (93.9) 57 (91.9)	35 (6.1) 5 (8.1)	0.555
Cardiac disease No Yes	310 (95.4) 282 (91.9)	15 (4.6) 25 (8.1)	0.069
Foot problems No Yes	437 (95.2) 155 (89.6)	22 (4.8) 18 (10.4)	0.010
Dizziness No Yes	484 (95.7) 108 (85.7)	22 (4.3) 18 (14.3)	<0.001
Cancer No Yes	561 (93.8) 31 (91.2)	37 (6.2) 3 (8.8)	0.539
Hearing Good Poor	422 (94.8) 170 (90.9)	23 (5.2) 17 (9.1)	0.065
Vision Good Poor	543 (93.5) 49 (96.1)	38 (6.5) 2 (3.9)	0.461
Urine control Good Poor	457 (94.6) 135 (90.6)	26 (5.4) 14 (9.6)	0.079

SOMCT = Short orientation Memory Concentration Test score (measure on a continuous scale); Secs=seconds; P-value: χ^2^ test.

**Table 3:** Association of physical assessments and measurements at baseline with recurrent falls at follow-up

Characteristic	Single or no fall	Recurrent falls	P Value
Age	73 (69 – 78)	73 (70–79)	0.387
Total number resident in dwelling	3 (2 – 6)	2 (1 – 4)	0.006
Socio-economic status index (SES)	7 (6 – 8)	7 (7 – 8)	0.423
Geriatric depression scale score (GDS)	2 (1 -4)	2 (1 – 5)	0.022
Cognitive score (SOMCT)	4 (0 – 8)	6 (2 – 10)	0.067
Number of comorbidities	3 (2 – 4)	4 (3 – 6)	0.015
Total Number of drugs	4 (2 – 6)	5 (3 – 7)	0.016
BMI (kg/m^2^)	28 (25 – 32)	25 (23 – 30)	0.100
Hand grip (kg)	16 (11 – 21)	13 (5 – 20)	0.019
One leg stand (eyes open) (seconds)	9 (4 – 23)	11 (4 – 25)	0.988
One leg stand (eyes closed) (seconds)	3 (2 – 6)	3 (1 – 8)	0.771
Timed Up & Go (seconds)	14 (11 – 20)	17 (11 – 25)	0.069
Chair stands (seconds)	14 (11 – 20)	8 (12 – 19)	0.112

Data are medians and interquartile range. SES=socio-economic status index: score of 8 items in household; Kg=kilogram; SOMCT = Short orientation Memory Concentration Test score (measure on a continuous scale); Mann-Whitney test.

Risk factors reported at baseline that were significantly associated with recurrent falls at follow-up include: area of residence, history of a previous fall, perceived health compared to a year ago, self-rated mobility, certain self-reported medical conditions (stroke, depression, foot problems (ulcers, bunions and callouses) and dizziness), and the number of residents in a dwelling, geriatric depression scale score, numbers of comorbid conditions, total number of drugs taken and hand grip strength. On logistic regression analysis, the variables significantly associated with recurrent falls were: history of a previous falls (OR 9.26; 95% CI 4.18–20.83), p<0.001); self-reported poor mobility (OR 3.30; 95% CI 1.54–7.07), p=0.002; and self-reported dizziness (OR 2.52; 95% CI 1.23–5.13), p<=0.011. The risk for recurrent falls increased nine-fold for those with a history of a previous fall. All the assessments and measurements had no association with recurrent falls (Table 4).

**Table 4:** Stepwise logistics regression analysis for baseline factors associated with follow-up recurrent falls.

Factor	OR	95% CI	P Value
History of previous fall	9.26	4.18 –20.83	<0.001
Self-reported poor mobility	3.30	1.54 – 7.07	0.002
Self-reported dizziness	2.52	1.23 – 5.13	0.011

Referent group is single or non-fallers vs. recurrent falls. OR=odds ratio, CI=confidence interval. P-value: Wald test

## Discussion

Several methodological challenges were encountered and had to be overcome in the study to establish prevalence rates for falls and recurrent falls, and to identify risk factors for falls in the older population. The challenges and how they were met may be anticipated in such studies in other low- to middle-income country settings, and our description thereof may be instructive for investigators planning similar studies in such settings.

A decision to draw a multi-ethnic study to compare data for three sub-samples was fortuitous; markedly different rates established for the sub-samples raise questions of their own. Such questions pertain to the role of socio-economic factors, as well as other indicators of disadvantage or deprivation across the ethnic groups within South Africa's politico-historical context on fall rates and risks for falls. The black African sub-sample reported the lowest number of falls (6.4%) and the white sub-sample the highest number (42.9%), with the coloured sub-sample falling in between (34.4%), closer to the rate for whites than for black Africans. Preliminary conclusions drawn to explain the differences pertain to the presence of comorbid conditions and occupational history (life-time manual versus non-manual labour) — the majority of black Africans having engaged in manual labour.

Key logistical challenges in the execution of the study included the following: Gaining access to the community of two suburbs (Gugulethu and Plumstead), initially problematic, resulted in delays which extended the study's time frame and impacted the budget. In the black African community of Gugulethu, or indeed any predominantly black African township in South Africa, cultural norms require researchers to obtain community leaders' permission, typically male elders, to carry out research in the area. Gatekeepers to the townships tend to be wary of any initiative that may exploit residents and want assurance on how the study outcome will benefit them and the community as a whole. Once satisfied, and they give permission, the leaders will inform the township community accordingly, which will help to publicise the activity and to improve the field workers' access to community members.

It behoves researchers thus who aim to work in such settings to meet requirements such as the foregoing in order to facilitate execution of a study in the area. Unnecessary delays, such as waiting for permission from gatekeepers, impact a study's time frame. In cases where previous studies are lacking, it may be necessary to conduct a pilot study to calculate a sample size. Where special equipment is needed to measure performance, the equipment will need to be validated. Conducting such studies prior to a baseline has implications for a study's budget.

In turn, a limited research budget and a resource poor study setting can necessitate methodological decisions, which was the case in the present study. On the other hand, fortuitously, physical assessment of subjects in their dwelling, instead of at a central venue, made it possible to include subjects who were frail and/or home bound, and would otherwise have been excluded from the study. A consequent increase in the case-mix thus served to reduce bias in the study findings.

The Cape Town study was designed to obviate a need for subjects to record fall events (e.g. in a diary) between baseline and follow-up — the conventional procedure in the gold standard prospective research design^20^. Several retrospective studies have reported subjects' recall of an event such as a fall as a particular methodological challenge^29^. Investigators in study communities with low literacy have reported difficulties in this regard. In Nigeria, Bekibele and Gujere^30^ found an average national literacy rate of 58 per cent for women and 78 per cent for men. In Rwanda, Ntagungira^31^ met subjects personally to set up appointments for interviews, as no telephonic communication was available. In China, Chu and others^32^ found 51 per cent of their subjects lacked formal education and were unable to record events on calendars, which the authors contended impacted the quality of the data collected. In the present study, low literacy levels in the black African sub-sample, and to a lesser extent in the coloured sub-sample, as well as a lack of universal access to a telephone made it impractical to use the gold standard research design for prospective studies on falls, which entails frequent communication with study participants to monitor and encourage the recording of events.

Attrition of subjects between baseline and follow-up constituted a logistical challenge. A high attrition rate is expected in any follow-up or longitudinal study in the urban black African population of South Africa. The high attrition rate of 24.5% in the present study may be due to factors such as black Africans not being indigenous to the Western Cape Province, and the majority migrating there from the Eastern Cape Province. A circular migration pattern prevails among black African in-migrants to Cape Town: the majority return to their ancestral homestead in a rural area of the Eastern Cape for a period once or twice a year, particularly over the Christmas recess. Half of the subjects (50%) in the study lost to follow-up were black Africans and it conjectured that circular migration may have contributed to the attrition. Investigators who conduct studies on a highly mobile population should thus factor in possible high attrition in the calculation of a sample size.

Resource limitations in the present study (the relatively modest budget and numerous subjects' lack of access to a telephone) were partially overcome through the study design. Nonetheless, a conceivable weakness of the study may lie in its cross-sectional design, which provided for a single follow-up visit and data collection at an end point. As a consequence, the prevalence and incidence rates of falls reported may be an under estimate of the true rates because of poor subject recall. Nonetheless, the study and its findings as a function of the adaptive strategies employed in the research design may be viewed as both affirmative and serendipitous. First, the fall prevalence rate of 26.4% in the baseline survey correlates strongly with the rate established in the pilot study. Second, the differential prevalence rates for the three ethnic sub-samples raise interesting questions for future investigation as to which factors may contribute to the differences - in particular, the markedly low fall rate for black Africans despite pronounced socio-economic disadvantage. Whereas poor mobility and dizziness would increase the risk to falls, particularly recurrent falls, a risk factors for falls such history of a previous fall, and ethnicity as found in this study may not be a risk factor for falls per se, but a proxy for multiple interacting physical, mental, cultural and life style factors that collectively contribute to a fall in the presence of an environmental precipitant. Finally, the study was conducted in an urban based, non-institutionalised older population; additional challenges might be anticipated in a rural based study population. Distances in rural areas are vast and public transport is sparse. Literacy levels of older study participants in rural areas are likely to be lower than in urban communities. In South Africa, and indeed elsewhere in the sub-continent, dwellings in rural areas may take the form of mud huts, with uneven floors, unsuitable for assessment of physical performance. Recruitment, training and supervision of field workers in these areas may prove challenging due to a lower educational levels and a lack of field work experience.

Strengths of this study lie in the community-based setting and performance of physical assessments in subjects' dwellings, which enabled the inclusion of frail and/or house bound subjects who would not have been able to travel to a central venue for assessment. An increased case-mix reduced bias in the study findings.

**Limitations of the study were:** that characteristics of subjects who consented to participate may have differed from those of non-participants; the non-participants may not have been prone to a fall. The cross-sectional nature of data collection both at baseline and at follow-up was subject to recall bias of a fall event and hence possible under estimation of fall prevalence. A lack of censored data on those lost to follow-up impacted the findings, as their characteristics may have differed from those who were followed up.

## Conclusion

Researchers who employ the gold standard prospective research design in studies on falls in an older population of a low to middle-income country should anticipate methodological challenges and devise adaptive strategies to overcome the challenges. Economic constraints in South Africa and the socio-economic heterogeneity of the multi-ethnic population pose several challenges for researchers. The experience reported here may be instructive to investigators who plan a study on falls in an older population in a similar country setting.
